# Male partner involvements in PMTCT: a cross sectional study, Mekelle, Northern Ethiopia

**DOI:** 10.1186/1471-2393-14-65

**Published:** 2014-02-12

**Authors:** Fisaha Haile, Yemane Brhan

**Affiliations:** 1 College of Health Sciences Department of Public Health, Mekelle University, Mekelle, Ethiopia; 2 Addis Continental Institute of Public Health, Addis Ababa, Ethiopia

**Keywords:** Male and PMTCT, Male and ANC, Male and HIV, HIV and ANC

## Abstract

**Background:**

Male partner participation is a crucial component to optimize antenatal care/prevention of mother to child transmission of HIV(ANC/PMTCT) service. It creates an opportunity to capture pregnant mothers and their male partners to reverse the transmission of HIV during pregnancy, labour and breast feeding. Thus involving male partners during HIV screening of pregnant mothers at ANC is key in the fight against mother to child transmission of HIV(MTCT). So, the aim of this study is to determine the level of male partner involvement in PMTCT and factors that affecting it.

**Methods:**

A Cross-sectional study was conducted among 473 pregnant mothers attending ANC/PMTCT in Mekelle town health facilities in January 2011. Systematic sampling was used to select pregnant mothers attending ANC/PMTCT service after determination of the client load at each health facility. Clinic exit structured interviews were used to collect the data. Finally multiple logistic regression was used to identify factors that affect male involvement in ANC/PMTCT.

**Results:**

Twenty percent of pregnant mothers have been accompanied by their male partner to the ANC/PMTCT service. Knowledge of HIV sero status [Adj.OR (95% CI) = 0.43 (0.18- 0.66)], maternal willingness to inform their husband about the availability of voluntary counselling and testing services in ANC/PMTCT [Adj.OR (95% CI) =3.74(1.38-10.17)] and previous history of couple counselling [Adj.OR (95% CI) =4.68 (2.32-9.44)] were found to be the independent predictors of male involvement in ANC/PMTCT service.

**Conclusion:**

Male partner involvement in ANC/PMTCT is low. Thus, comprehensive strategy should be put in place to sensitize and advocate the importance of male partner involvement in ANC/PMTCT in order to reach out male partners.

## Background

Male partner participation is a crucial component in the optimization of PMTCT service. For this reason, antenatal care /prevention of mother to child transmission (ANC/PMTCT) is the only opportunity to capture pregnant mothers and their male partners to prevent the transmission of HIV during pregnancy, labour and breast feeding [1]. Because More than 90% of childhood HIV infections are due to mother to child transmission thus 600,000 children are newly infected by HIV worldwide annually. Current estimate suggests that [2,3].

Acquired immune deficiency syndrome (AIDS) has now become the leading cause of under five deaths in sub Saharan Africa. It has further worsened the morbidity and mortality of infants and children, pertaining to its MTCT [4]. So, PMTCT programs provide an opportunity for both prevention of HIV transmission from mother to child and enrolment of infected pregnant women and their families into antiretroviral treatment [5,6].

Involvement of male partners may increase adherence to PMTCT and its program outcomes [7]. So, Male partner participation was associated with positive outcomes such as: greater use of Antiretroviral therapy, higher acceptance of post-test counselling among pregnant women ,increased spousal communication about HIV and safe sex. Additionally, detection of maternal infection in early pregnancy through provider initiated HIV testing and counselling (PITC) is not enough to mitigate mother to child transmission of HIV but only few husbands accompany their wives to the ANC/PMTCT clinic. Consequently ,involving male partners in ANC/PMTCT is very crucial in the fight against transmission of the virus to children [8,9].

In addition to the above points, involving male partners in ANC/PMTCT can often be utilized as an entry point for the provision of additional PMTCT services notably partner testing, condom use and infant feeding recommendations for both male and female participants [10]. But, Only 18% of women were accompanied by their partners for testing and counselling among women counselled as a couple. This is the result of narrow focus of PMTCT to date which represents a lost opportunity to effectively combat the vertical transmission of HIV to children – a largely preventable infection given current scientific knowledge [11].

The second reason that rationalize male partner involvement in ANC/PMTCT is that there is an over growing discordant rate among couples. In Ethiopia, HIV prevalence among cohabiting individuals is notably high in urban areas (10.9%); of whom about 72% (i.e. 7.8% of the total) of the cohabiting couples are discordant. In urban areas, 5.6% of HIV negative married men are living with infected wives and 2.2% of Married HIV negative women are living with infected husbands [12]. This witnesses that, screening the mother only to prevent mother to child transmission of HIV doesn’t safeguard the child from acquiring HIV. For this reason, for effective PMTCT interventions, male partners should be involved in their Wives’ ANC/PMTCT care [13]. Moreover, there are limited studies about level of male involvement and associated factors. So this study will try determining the level of male involvement in ANC/PMTCT service and to identify factors that affect male involvement in ANC/PMTCT service.

## Methods

### Study settings

Mekelle is a town located 784 kms far north of Addis Ababa. It has a total population of 215, 546 with a sex ratio of 95 males to 100 females. Pregnant mothers constitute 3.7% of the the total population. The town health office structure is made up of 10 health facilities, with all of them offering ANC and PMTCT service.

### Design, participants and sampling procedure

Facility based cross sectional study was done in January 2011. Data was collected from 473 pregnant mothers who were attending antenatal care (ANC/PMTCT) at five randomly selected Health Facilities (4 health centers and 1 hospital) in Mekelle, northern Ethiopia. The source population was all pregnant women attending antenatal care in public health facilities since these health facilities serve the majority of the population in the town’s antenatal care (ANC/PMTCT) service especially the poor Population and the study population was all pregnant women attending antenatal care during the Data collection period.

Sample size was determined using the formula for two population proportion estimation using Epi-info stat calc for un matched case control with the assumption of 95% certainty level, 5% precision, and 80% power, 4:1 control to case ratio and considering the odds ratio to be detected between the two groups as 2.0. Like wise, the required sample size was 95 cases (mothers who bring their male partner to the ANC/PMTCT) and 378 controls (mothers who didn’t bring their husband to the ANC/PMTCT. Stratified sampling technique with proportional to size was used to select the study participants. Finally, the determined sample for each health institution was achieved through exit interview from systematically sampled and voluntarily consented pregnant women with in four weeks of working days. To avoid double counting each card of interviewed mothers was marked using red marker.

### Data collection and quality control

Clinic staffs providing ANC services were trained on the data collection and Interview techniques. Interviewer administered structured questionnaire was used to collect the data. The designed questionnaire was translated first into the local language, Tigrigna and back translated to English to ensure its consistency. The questionnaires were pretested in similar settings two weeks before the data collection and it was used to elicit the following information from the study participants: characteristics of pregnant women and male partners and proximate determinants of male involvement in ANC/PMTCT. The completeness and consistency of data was assured through direct and daily supervision by the supervisor and principal investigators. Data coding and cleaning were performed by cross-Checking the printout data for obvious errors to assure quality of data.

### Statistical analysis

Data was entered into Epi-Info software version 3.5.2, edited, cleaned and analyzed using SPSS version 16.0. Male involvement is determined by the proportion of male partners accompanying their pregnant wives during ANC/PMTCT service. Additionally, descriptive statistics such as frequencies and proportion was used to describe the study population in relation to relevant variables. Explanatory variables found to be statistically significant in bivariate logistic regression analysis were entered into multiple logistic regression analysis (backward stepwise method) for adjustment of confounding effect between independent variables (Age, Educational status of mothers and their male partner, house hold income, previous knowledge of HIV status, marital status, duration of relationship, male partner as the first person to share HIV test result, ever tested and counseled together, husband informed about the availability of VCT in the ANC) and the dependent variable (Male involvement). Odds ratio with 95% confidence interval were computed to assess the presence and degree of association between the dependent variable (male partner involvement) and independent variables (Current knowledge of her sero status, Male partner as the first person to share HIV status, ever tested and counselled with her husband, Husband informed about the availability of VCT at the ANC/PMTC).

### Ethical consideration

Before conducting the study Ethical clearance was obtained from the institutional review board (IRB) of Addis Continental Institute of Public Health and Mekelle University. Permission to conduct the study in each health facilities was secured from the respective Health institutions in Mekelle Town. Verbal informed consent was obtained from each study participants after clear explanation about the purpose, benefit and risks of the study.

## Results

### Demographic characteristics of participants

A total of 473 pregnant mothers attending ANC/PMTCT were interviewed in selected health facilities of Mekelle town and all of them fully responded to the questionnaire. The majority 402(85.0%) of the respondents were mothers with formal education. The mean number of years attended in school for mothers who were accompanied by their male partners and mothers who didn’t were up to grade nine and eight respectively with equal standard deviation of four years. Most pregnant mothers were attended 1^st^ and 2^nd^ ANC visit 324(68.5%). Large number 317(67.0%) of pregnant mothers were married and the average duration of relationship in years with their current male partner for mothers who were accompanied by their male partners and who didn’t, were 4.3 ± 4.0 and 5.3 ± 4.4 years respectively (Table 1).

**Table 1:** Socio demographic characteristics of pregnant mothers attending ANC/PMTCT, Mekelle Town, Northern Ethiopia, 2011

**Variable**	**Mother accompanied by her partner**		**Total**
**Age**	**Yes, N = 95**	**No, N = 378**	
15-19	15(15.8)	38(10.1)	53(11.2)
20-24	38(40.0)	116(30.7)	154(32.6)
25-29	28(29.5)	136(36.0)	164(34.7)
> = 30	14(14.7)	88(23.3)	102(21.6)
**Level of education**			
Unable to read and write	10(10.5)	59(15.6)	69(14.6)
Able to read and write	4(3.4)	13(4.1)	17(3.6)
Primary education	34(35.8)	165(43.7)	199(42.1)
Secondary education	32(33.7)	100(26.5)	132(27.9)
Post secondary education	15(15.9)	41(10.8)	56(11.8)
**No of ANC visits**			
One visit	27(28.4)	131(34.7)	158(33.4)
Two visit	35(36.8)	131(34.7)	166(35.1)
Three visit	22(23.2)	69(18.3)	91(19.2)
Four and above	11(11.6)	47(12.4)	58(12.3)
**Marital status**			
Currently married	70(73.7)	247(65.3)	317(67.0)
Living with a man but not married	23(24.2)	90(23.8)	113(23.9)
Not living together but not separated	2(2.1)	41(10.8)	43(9.1)
**Occupation**			
No occupation	58(61.1)	256(67.8)	314(66.4)
Government employee	37(38.9)	122(32.3)	159(33.6)

### Male partner involvement in ANC/PMTCT and factors that affect it

Only 95/473 (20.1% with 95% CI (16.7%, 23.9%)) were accompanied by their male partner at the ANC/PMTCT. Among male partners who accompany their pregnant wives, 78(82.1%) with 95% CI (73.4%, 88.9%) of husbands who accompany their pregnant wives have tested and counseled for HIV in the current pregnancy of their wives at ANC or show their test results done else where. Pregnant mothers who know their sero status were less to bring their partner to the ANC/PMTCT clinic than those who didn’t, Adj.OR = 0.4. However, mothers who have ever been tested together with their male partner were five times higher to bring their husband to the ANC/PMTCT clinic than those who have never been tested and counselled together with their male partner. More over, pregnant mothers who inform their male partner about the availability of VCT at the ANC were four times higher to be accompanied by their partner than their counter parts (Table 2).

**Table 2:** Association between Male partner involvement in ANC/PMTCT and each explanatory variable (Crude & adjusted OR), Mekelle, Northern Ethiopia, 2011

**Variable**	**Mother accompanied by her husband**		**COR with 95% CI**	**AOR with 95% CI**
	**Yes n = 95**	**No n = 378**		
	**No (%)**	**No (%)**		
**Maternal age**				
15-19	15(15.8)	38(10.5)	2.5(1.1,5.6)	2.2(0.7,6.3)
20–24	38(40.0)	116(30.7)	2.1(1.1,4.0)	1.8(0.74,4.5)
25–20	28(29.5)	136(36.0)	1.3(0.7,2.6)	1.1(0.5,2.8)
> = 30	14(14.7)	88(23.3)	1.00	
**Maternal educational status**				
No formal education	10(10.5)	59(15.6)	1.00	2.7(0.6,12.3)
Read and write	4(4.2)	13(3.4)	1.82(0.5,6.7)	1.3(0.5,3.4)
Primary education	34(35.8)	165(43.7)	1.21(0.6,2.6)	1.9(0.7,5.3)
Secondary education	32(33.7)	100(26.5)	1.89(0.9,4.1)	3.09(0.96,9.9)
Post secondary	15(15.8))	41(10.8)	2.16(0.9,5.2)	
**House hold income**				
< = 500	16(16.8)	49(13.0)	1.00	
501–1000	32(33.7)	147(38.9)	0.68(0.34,1.3)	0.58(0.19,1.2)
> = 1001	47(49.5)	182(48.1)	0.79(0.4,1.5)	0.7(0.36,1.6)
**Duration of relationship(in yrs)**				
<5	59(62.1)	191(50.5)	1.00	
5–10	25(26.3)	132(34.9)	0.61(0.37,1.0)	0.5,(0.23,1.1)
10 and above	11(11.6)	55(14.6)	0.7(0.3,1.3)	0.5(0.21,1.0)
**Marital status**				
Not in union but not separated	2(2.1)	41(10.8)	1.00	
Living with a man but not married	23(24.2)	90(23.8)	5.2(1.2,23.3)	2.6(0.54,12.5)
Currently married	70(73.7)	247(65.3)	5.81(1.4,24.6)	2.7(0.59,12.5)
**Husband’s Educational status**				
No formal education	4(4.2)	39(10.30	1.00	
Primary education	34(35.5)	106(28.0))	3.1(1.0,9.4)	1.8(0.52,6.2)
Post primary	57(60.0)	233(61.6)	2.4(0.8,7)	1.2(0.32,4.2)
**Knowledge of their sero status**				
Know her sero status	93(97.9)	353(93.4)	0.4(0.25,0.8)	0.4(0.18,0.7)**
Didn’t know her sero status	2(2.1)	25(6.6)	1.00	
**Male partner as the first person to share HIV test results**				
Yes	2(2.1)	353(93.4)	3.3(0.8,14.2)	1.4(0.28,6.6)
No	93(97.9)	25(6.6)	1.00	
**Ever tested and counseled with their husband**				
Yes	83(87.4)	208(55.0)	5.7(3.0,10.7)	4.7(2.32,9.4)**
No	12(12.6)	170(45.0)	1.00	
**Husband informed about the availability of VCT at the ANC**				
Yes	0(94.7)	292(77.3)	5.24(2.06,13.3)	3.74(1.38,10.2)**
No	5(5.3)	86(22.7)	1.00	

**Statistically significant at P < 0.05.

## Discussions

In this study, the level of male involvement in ANC/PMTCT was low at 20.1% (16.7%, 23.9%) but a little higher than studies done in Kampala and Mable district, Uganda and Nairobi, Kenya. The studies done in Kampala and Mable district, Uganda showed that male involvement in the different PMTCT activities was 16.5% and 4.5% respectively [13,14]. Similarly a study conducted at Nairobi antenatal clinic, Kenya revealed that male partner participation in antenatal volunteer testing and counselling with their spouses was low [15]. This difference could be attributed to variations in setting and defining the term male involvement in ANC/PMTCT.

Of the 95/473(20.1%) of male partners who accompany their pregnant wives, 78(82.1%) of them have been tested for HIV. This is higher when compared with a study done in Cameroon which reported that 147(58.7%) of husbands had at least one HIV test at ANC [16]. Most 147(58.7%) of pregnant mothers have been encouraged by their male partners to go for ANC but 157(33.2%) of the respondents think that husbands who accompany their pregnant wives to the ANC/PMTCT clinic were bewitched. Similar study from Cameroon reported that 114(45.2%) of men think that the community sees male partners who accompany their wives to the ANC/PMTCT as being jealous and over protective of their wives [16]. The reasons for not being accompanied by their male partners were fear of violence and divorce if tested positive in front of their husband. This was similar with a study done in Mabeya Region, Tanzania which reveals that the negative events in case, the partner came along with for HIV testing and counselling and they would test positive will be in fear of violence and divorce [17].

Considerable number, 108 (22.8%) of mothers point out that ANC/PMTCT services were for pregnant women and children only compared with a study done in Lusaka, Zambia, which reported that only 10% of mothers were able to encourage their husband’s participation in ANC/PMTCT [18]. Significant number 67(14.2%) of pregnant mothers stated that they should consent their male partner about HIV testing and counselling at ANC. This was very low when compared with a study done in Cameron which reported that 100(81%) of men thought that pregnant mothers should consent their husband for ANC testing and counselling for HIV at ANC [17].

Half 239(50.5%) of male partners arrange transport and provide financial support to their pregnant wives to go for ANC/PMTCT. This finding was similar with a study done in Cameron where 64.7% of male partners provide financial support for their pregnant wives to go for ANC/PMTCT service [16].

This study revealed that, 97.5% of participants indicated that they were ready to disclose their test results and suggest HIV testing and counselling for HIV to their male partner. Similar study study done in Dominican Republic Vietnam indicates that 90% of pregnant mothers have been disclosed and suggest HIV testing and counselling to their male partner [19,20].

Factors which affect pregnant mothers to bring their male partners to the ANC/PMTCT clinic were: maternal knowledge of sero status, previous couple counselling and knowledge of the male partner about the availability of voluntary counselling and testing service at the ANC clinic. Mothers who knew their sero status were 0.4 times less to bring their male partners to the ANC/PMTCT clinic. But a study done in Mbale district, Uganda depicted that mothers who know their HIV sero status were 4 times heigher to get involved in PMTCT services than those who didn’t know [18]. This difference could possibly be due to the perception of pregnant mothers in this study that their HIV testing and counselling was proxy to their husband’s HIV testing and counselling.

Previous testing and counselling for HIV was also associated with increased husband accompaniment of pregnant mothers to the ANC/PMTCT clinic. Respondents who have been tested and counselled with their husband previously were 5 times higher to come with their husbands than their counter parts. Being informed about the availability of VCT service in the ANC was also associated with increased husband accompaniment i.e. mother who tell their male partners about the availability of VCT service in the ANC clinic were 4 times higher to be accompanied by their male partner to the ANC than those who didn’t. The same study done in Uganda showed that increased knowledge and awareness of male partners about the availability of VCT services in the ANC clinic and its importance with the prevention of mother to child transmission of HIV was associated with increased husbands participation in ANC/PMTCT [13,21].

### Study limitations

This study assesses male involvement in the perspective of pregnant wives which may affect the reliability of information about male characteristics like, age, education and HIV test. More over, one parameter (presence of male partner in the ANC) was used to measure male involvement in ANC/PMTCT which may underestimate male involvement. The other limitation of this study was that since this study was done in ANC clinics in urban areas, it may not be applicable in rural settings.

## Conclusion

In conclusion the level of male partner involvement in ANC/PMTCT were low. The factors that affect pregnant mothers to accompany their male partners were maternal knowledge of HIV sero status, maternal willingness to inform their husband about the availability of VCT services at ANC and previous history of couple counselling are independent predictors of male participation in ANC/PMTCT. As result, comprehensive strategy to sensitize and advocate the greater importance of male partner involvement in ANC/PMTCT needs to be developed to reach out male partners.

## Competing interests

The author(s) declare that they have no competing interests.

## Authors’ contributions

FH involved in proposal writing, designing, and recruitment and training of Supervisors and data collectors, analysis and write-up and in all stages of the project implementation. He did most of the analysis and write up of the paper. YB contributed in the designing of the methodology, lead investigator and involved in designing of project proposal, design of questionnaires, supervision and involved in the analysis stage of the project and final approval of the paper. Both authors read and approved the final manuscript.

## Pre-publication history

The pre-publication history for this paper can be accessed here:

http://www.biomedcentral.com/1471-2393/14/65/prepub
